# Effect of mounting a sound suppressor on distribution and total amount of inorganic gunshot residue on targets

**DOI:** 10.1111/1556-4029.70025

**Published:** 2025-04-03

**Authors:** Matteo Donghi, Alessandro Girella, Debora Pellegrino, Federica Maraschi, Antonella Profumo, Chiara Milanese, Daniele Merli

**Affiliations:** ^1^ Arma Dei Carabinieri, RIS Parma Parma Italy; ^2^ Dipartimento di Chimica Università Degli Studi di Pavia Pavia Italy; ^3^ INFN Sezione di Milano‐Bicocca Milan Italy

**Keywords:** gunshot residue GSR, inductively coupled plasma optical emission spectroscopy ICP‐OES, silencer, sound suppressor, x‐ray fluorescence spectroscopy XRF

## Abstract

Sound suppressors critically modify barrel overall length and ballistic performances of hosting guns and are therefore expected to influence the patterns of the plumes of gunshot residues (GSR) reaching the targets. Despite the forensic interest, in recent years, a single paper was published on the variations induced by the use of a suppressor in the spatial distribution of visible soot clouds on close targets and in the number of a few selected classes of GSR particles detected by scanning electron microscopy. A different approach, based on x‐ray fluorescence (XRF) and inductively coupled plasma‐optical emission (ICP‐OES) spectroscopies, that points to most of the metallic elements ejected from the barrel, is suggested here. The confirmed effect of a sound suppressor up to a distance of 20 cm is to gather the cone of particles reaching the target. Despite this pattern modification, the global concentrations of the ammunition‐related elements of interest (Pb, Ba, Sb, Cu) on targets cannot be considered significant (i.e., with 95% confidence) reduced by the use of a suppressor, due to the high intra‐specimen and inter‐specimen ICP‐OES data dispersions. Differently, the hypothesis of a role of homemade suppressors in enriching GSR populations in Fe is supported by our results. The presence of iron is indeed the only indication of the actual use of a suppressor, a piece of information that is useful for the correct interpretation of the lead patterns visualized on targets for muzzle‐to‐target determination.

**Highlights**

- The effects of a sound suppressor on the GSR plume ejected by a firearm were investigated.
- XRF was used as a quantitative, non‐destructive technique to determine the firing range.
- XRF maps showed that suppressors concentrate GSR patterns up to a range of 20 cm.
- ICP‐OES detected no statistically relevant variation in Pb–Ba–Sb–Cu concentrations on the targets.
- Suppressor‐induced Fe presence in GSR on targets was detected for shooting distances of up to 40 cm.

## 1 INTRODUCTION

Gunshot residue (GSR) collection and forensic analysis can play a role in criminal investigations when a firearm has been discharged, providing useful elements for reconstructing shooting incidents [1, 2]. To date, however, some interpretation difficulties remain, mainly due to the high variability of GSR specimens. Indeed, the total amount, distribution, and composition of the recovered GSR particulate depend not only on the nature of the discharged firearm and ammunition [3, 4, 5] but also on the physical properties of the target surface [6, 7], its distance from the muzzle, and, more generally, the position of the residue collection point around the firing weapon [8, 9, 10].

The so‐called “memory effect in weapons,” that is, the possibility to recover residues whose composition depends on the shooting history of the firearm and not only on the last round, is probably the most investigated example of the direct influence of the gun on the composition of the GSR population [11, 12, 13, 14]. Firearm surface treatments, the presence of specific lubricants or rust in the barrel, and the use of some accessories such as sound suppressors [15, 16, 17, 18] are other firearm‐related factors influencing the chemical nature of inorganic gunshot residue. Among the cited examples, the use of sound suppressors is the most peculiar, as it is supposed to affect not only the elemental profile of emitted GSR particles but also their spatial distribution [18].

Suppressors, fully integrated into guns or simply attached to barrels as disposable accessories, are often associated with the criminal use of firearms. A suppressor is a device designed to reduce both the visible flame (flash suppression) and the noise (sound suppression) generated by a shot, usually for tactical or sporting purposes [19]. Due to their capability to reduce the sharp sound at the muzzle, suppressors are commonly referred to as silencers [20], although other shooting‐related noises, such as the sonic boom of the bullet and any mechanical clack caused by the reloading process [21], remain unaffected by their use. A suppressor typically consists of two coaxial metal cylinders: the inner, slightly wider than the caliber of the firearm, has a drilled surface to allow hot gases to expand into the internal chambers of the outer cylinder [19]. Expansion chambers, often filled with inert material, are designed to reduce the temperature and the outgoing velocity of the propellant gases, in accordance with the first law of thermodynamics. The plume of metallic particles ejected from the firearm is expected to suffer the same decrease in average velocity, with gunshot residues experiencing a shorter range of projection and a reduced ability to adhere to target substrates [18].

One determination which is often made is the evaluation of the distance between the target and the muzzle of the discharging weapon: by quantifying the amount and distribution of gunshot residues on targets [22], usually by chemo‐colorimetric tests to visualize the deposition patterns of nitrites from the ammunition gunpowder or of lead and copper from the projectiles [1], and the potential role of the suppressors should always be considered in comprehensive reconstructions of shooting incidents [23, 24].

Despite the widespread forensic interest, only one paper has recently been published on the effects of sound suppressors on the nature and distribution of inorganic GSR on targets [18]. The cited research documented a significant reduction of the visible soot cloud on close targets when a suppressor was coupled to a submachine gun and gave some insight into the GSR morphology by using SEM–EDX analysis. In detail, of all the inorganic particulate collected from targets and automatically classified by the GSR search routine, only spherical particles with one of the following compositions were counted: PbBaSb, BaSb, PbBa, PbSb, Pb, and Sb. This choice had two main disadvantages. Firstly, it was not possible to discriminate between different contributions to the total number of particles due to primer‐GSR or to residues originating from the bullets, the barrel, or the suppressor itself. In addition, thousands of features had to be manually checked for each sample to ensure not only that the morphological and compositional requirements were met but also that larger particles were only counted once. Despite the efforts made for data collection, no definitive results were reported. When cotton targets were used, a reduction in the number of residues recovered was documented for all but the shortest shooting distances (*d* ≤ 2 cm). In contrast, when pig skin was used as the target surface, the highest number of residues was found with the suppressor set at 14 cm, a value close to that previously measured for the unsilenced gun and cotton targets (*d* = 20 cm). The lack of supporting literature on the topic was reported as a limiting factor for data interpretation, and the author herself suggested considering the presented results only as descriptive of a general trend.

The aim of this study is to add the available experience and data on the effect of mounting a suppressor at the muzzle of a firearm on the residue population released. X‐ray fluorescence (XRF) and inductively coupled plasma‐optical emission (ICP‐OES) spectroscopies were preferred to scanning electron microscopy–energy dispersive x‐ray spectroscopy (SEM‐EDS) to analyze the inorganic GSR particulate retained by cotton targets hit at different shooting distances. Both these techniques allow quantitative data collection [25, 26, 27], without the need for particle‐by‐particle verification by the operator. Analyses are less time‐consuming, results are operator‐independent, and a wider list of chemical elements than Pb, Ba, and Sb can be investigated. Moreover, XRF is also a non‐destructive trace evidence tool [28] that can be used directly on victims' clothes, regardless of fabric, color, or presence of blood. For all these reasons, some forensic laboratories, mainly in Europe, have preferred XRF to the widely diffused chemographic coloring tests to evaluate the shooting distance [29, 30], despite the cost to purchase an x‐ray spectrometer.

## 2 MATERIALS AND METHODS

### 2.1 Firearm, ammunition, suppressor, and targets

**FIGURE 1:**
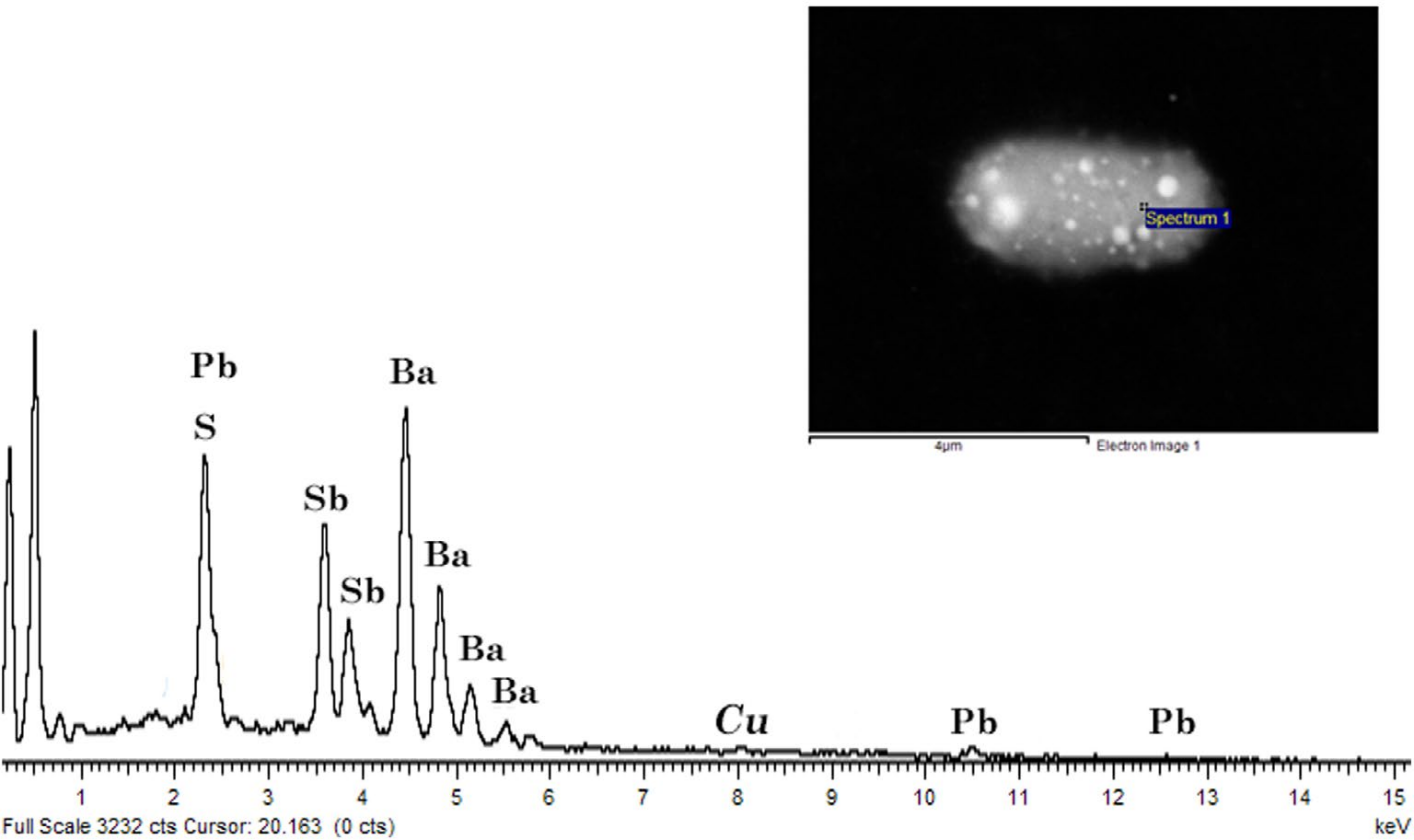
A typical gunshot residue from Fiocchi's primer mixture, collected from a cotton target after shooting. Further details on SEM‐EDS settings and description may be found in the Supporting Information.

A self‐reloading pistol, model 70S, caliber .32 ACP (7.65 × 17 mm Browning SR), by Beretta (Fabbrica d'Armi Pietro Beretta, Gardone V.T.–Italy) was used for the shooting tests. The firearm was loaded with .32 ACP cartridges by Fiocchi (Fiocchi Munizioni, Lecco – Italy), assembling 4.7 g (73 grains) FMJ bullets and using a type of SINOXID [31] primer mixture containing, according to the producer's informative card [32], barium nitrate (4%–6% w/w), lead styphnate (2%–4% w/w), antimony sulfide (1%–2% w/w), tetrazene (0.2%–0.4% w/w), and penthrite (0.3%–0.5% w/w). The characteristics of GSR particles from this ammunition are made of Pb, Ba, and Sb, usually with Ba as the main peak of the EDS spectrum and with the presence of S and Cu (see Figure 1).

**FIGURE 2:**
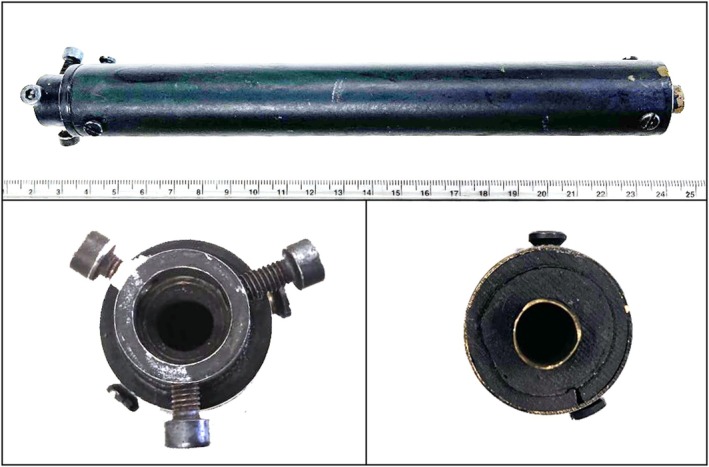
The homemade suppressor used for the test firings. Its production was completed the day before the shooting tests and it was dismantled immediately after them.

The suppressor was constructed using an inner drilled steel tube a few tenths of a millimeter larger in diameter than the barrel of the weapon and an outer aluminum tube, 20 cm long and 2 cm wide. Gas expansion chambers were filled using steel wool. The suppressor could be coupled to the part of the gun barrel protruding from the slide by means of three screws and a Teflon (PTFE) flange. The suppressor used for this study is reported in Figure 2.

Targets were made of uncolored (natural white) and untreated (neither washed nor ironed) cotton fabric. Each target was prepared according to an established procedure [7], consisting of clamping onto a rigid cardboard support a 20 cm × 20 cm square of cotton cut out of the fabric roll. After preparation, targets were stored separately in plastic bags to avoid any environmental contamination prior to shooting.

### 2.2 Shooting session details

A single shooter discharged all the rounds at an outdoor range (temperature: 18°C, wind speed: <5 km/h, humidity: 65%), using the above‐mentioned Beretta's pistol in both unsilenced and silenced configurations. Each target was hit by a single bullet. The following shooting distances were chosen: 5 cm, 20 cm, 40 cm, 60 cm, 80 cm, and 100 cm. Three replicas were made for each distance and pistol configuration, for a total number of 36 test specimens. Each target was then separately sealed into a plastic bag to avoid any possible cross‐contamination and returned to the laboratory for analysis.

Field blank targets were left exposed near the firing range but outside it throughout the shooting session. These control specimens were collected to verify the concentrations of elements of interest that could be recovered not as a result of direct gunshots but from the whole handling process of the cotton targets.

### 2.3 XRF mapping

A fluorometer model MIDEX II–LD by Ametek Spectro (Spectro Analytical Instruments GmbH, Kleve–Germany), equipped with a molybdenum‐anode tube (48 kV, 0.5 mA), was used to visualize the spatial distribution of metallic particles on targets, according to the operating procedure developed for shooting distance determination purposes [27, 29]. A working distance of 20 mm (in air) and a spot size of 2.4 mm on the specimen were used. With these parameters, a MIDEX II–LD fluorometer gives quantitative results for elements having atomic number *Z* > 21. For any element of interest, a quantitative distribution map can be plotted using a rainbow palette of colors.

An area of 150 mm × 150 mm around the bullet's hole of each test specimen was digitalized into a 68 pixel × 68 pixel matrix and analyzed using a dwell time (i.e., single‐pixel acquisition time) of 10 s. No preparation was required for the cotton targets to analyze.

### 2.4 ICP‐OES analyses

**FIGURE 3:**
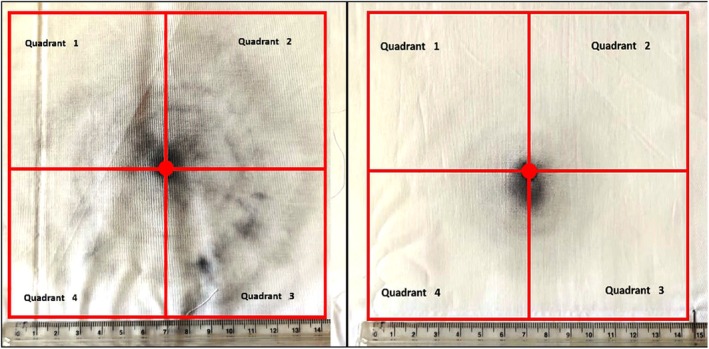
Cotton targets hit from a distance of 5 cm from the barrel muzzle (unsilenced gun, on the left) and from the suppressor muzzle (silenced gun, on the right). 7 cm × 7 cm squares used for ICP‐OES analyses are also shown and designated as quadrants 1–4.

For the ICP‐OES analyses, 14 cm × 14 cm squares, centered in the bullet hole, were cut out of the 20 cm × 20 cm targets and divided into four quadrants. Each analytical specimen was then a 7 cm × 7 cm cotton square, sharing a vertex at the bullet hole with the three other similar specimens. From all the specimens, the bullet hole areas were removed to avoid summing the bullet wipe contributions to the quantifications of residues of real interest, that is, those directly ejected from the barrel (see Figure 3).

Extractions of GSR were performed using a literature‐reported procedure [33]. Each specimen was soaked in 10 mL of 10% w/w nitric acid (from ultrapure 65% w/w HNO_3_, diluted in purified water produced by a Milli‐Q Direct system by Merck Millipore, Burlington, Massachusetts–USA) into a 50 mL polypropylene Falcon tube. The tubes were left overnight and then sonicated in an ultrasonic bath (at 70°C) for 30 min. Fifteen milliliter of Milli‐Q water were added to each tube to give a final volume of 25 mL. the fabrics were then removed, and extraction solutions were filtered through a 0.22 μm nylon syringe filter (diameter 2 cm, Whatmann, Cytiva, Marlborough–USA).

Element measurements were carried out by ICP‐OES by an iCAP 7400 Duo (Thermo Fisher Scientific Inc., Waltham–USA), equipped with a concentric nebulizer, a cyclonic spray chamber, and a ceramic duo‐torch, according to the operating conditions suggested by the manufacturer. An ASX‐560 autosampler (Teledyne CETAC, Omaha, Nebraska–USA) was used to transfer samples to the introduction system of the ICP‐OES. Thermo Scientific Qtegra™ software ver.2.8.2944.202 was used for data acquisition. Data from each specimen were processed without performing any background (field blank) subtraction because of its negligible values (see Section 3, paragraph 3.3).

The elements from Fiocchi's ammunition primer mixture (lead, barium, and antimony) and bullet jacket (copper), commonly found in IGSR particles from lead‐based primers, were selected for ICP‐OES measurements. Elements constituting the suppressor, iron and aluminum, were also considered for the analyses. The inorganic elements selected, that is, Pb, Ba, Sb, Cu, Fe, and Al, were quantified by an external standard calibration curve as follows. Standard solutions were prepared by dilution of ICP‐grade standards 1000 mg/L by Merck (Merck KGaA, Darmstadt – Germany) to 0.1 mg/L , 1 mg/L , 5 mg/L , and 10 mg/L and then acidified to a final concentration of 4% w/w nitric acid (from ultrapure 65% w/w HNO_3_) to cover the expected concentration range of analytes.

For each element, the method quantification limit (MQL), expressed as μg/cm^2^ of blank tissue, was calculated from the corresponding instrumental quantification limit (IQL) obtained from the calibration curve considering the whole extraction procedure (see Tables S1 and S2, where ICP‐OES wavelengths, measurement modes, IDL/IQL, and MDL/MQL are also reported). IQL for each element was defined as μ + 10σ, where μ is the average value of the element in the 4% w/w HNO_3_ solution and σ the corresponding standard deviation.

## 3 RESULTS AND DISCUSSION

### 3.1 Visual inspection

With the suppressor assembled to the pistol, a clear difference in soot blackening around the bullet hole was visible to the naked eye only for targets fired at a distance of 5 cm (see Figure 3). For longer distances (≥20 cm), the presence of the suppressor at the barrel muzzle did not cause any visible difference in the GSR deposits on the targets.

Our results differ from those presented by Brozek‐Mucha [18], who could still distinguish with the naked eye the residue patterns on cotton targets hit at a distance of 30 cm, whether the gun was silenced or not. Although the guns used in the two experiments are of the same caliber, non‐comparable cleaning conditions and different barrel lengths (115 mm for the CZ 61 submachine gun vs. 90 mm for the Beretta 70S pistol) could have played a role in the amount of residue deposited on targets.

### 3.2 XRF results

**FIGURE 4:**
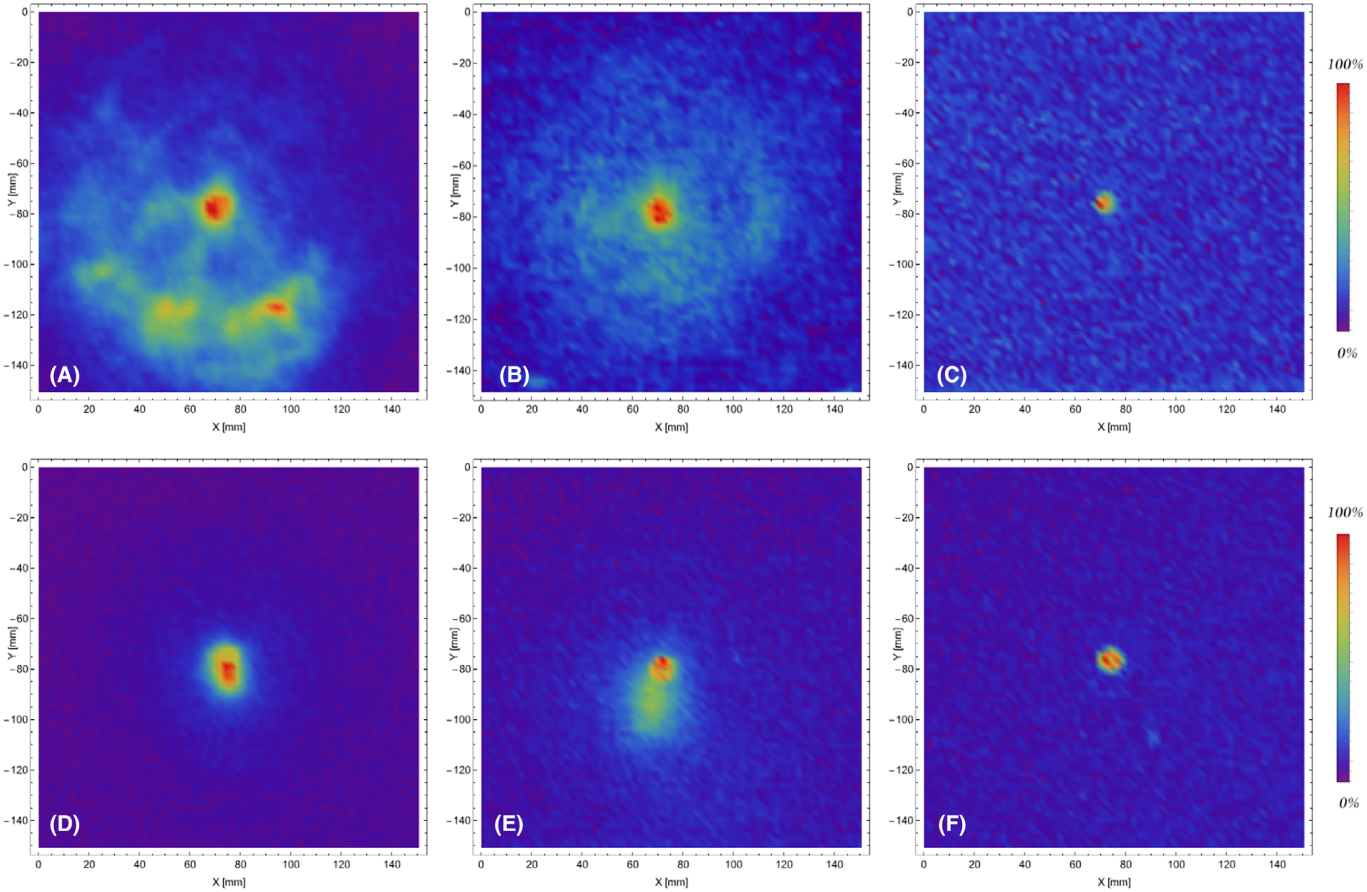
XRF maps of lead on cotton targets in rainbow false‐colors. In the upper row, specimens collected discharging the pistol without the suppressor, at the shooting distances of 5 cm (A), 20 cm (B), and 40 cm (C). In the lower row, specimens collected discharging the pistol with the suppressor mounted, at the shooting distances of 5 cm (D), 20 cm (E), and 40 cm (F). Area explored: 150 mm × 150 mm.

To show the effect of the suppressor on residue patterns on targets, XRF maps of lead on cotton targets for the shooting distances of 5 cm, 20 cm, and 40 cm are reported in Figure 4. Among all the chemical elements, lead was chosen because it is abundant in rifled bores fouling and in residues from the discharged ammunition (both the bullet and to a lesser extent to the primer); it is simply detected by x‐ray fluorescence spectrometry with no risk of misclassifications (due to the presence of the PbL emission lines at 10.55 keV, 12.61 keV, and 14.76 keV), and it evaporates easily, forming characteristic patterns on targets [34, 35] when the shooting distances are short enough (usually <40 cm).

The different lead patterns, which can be attributed to the presence of the suppressor, are clearly visible not only for the targets hit at the shooting distances of 5 cm but even at 20 cm. For these samples, the effect of the suppressor was to reduce the dimension of the ejection cone of the residues, which appear concentrated in a smaller area around the bullet's hole. At the longest shooting distances (≥40 cm), no lead patterns were detected on the targets but on the bullets' wipes despite the presence of the suppressor, as the ejection cones had become large enough to reduce the Pb fluorescence signal to the noise level. The dimensions, morphology, and intensity of the bullet wipe rings showed no variations induced by the suppressor.

**FIGURE 5:**
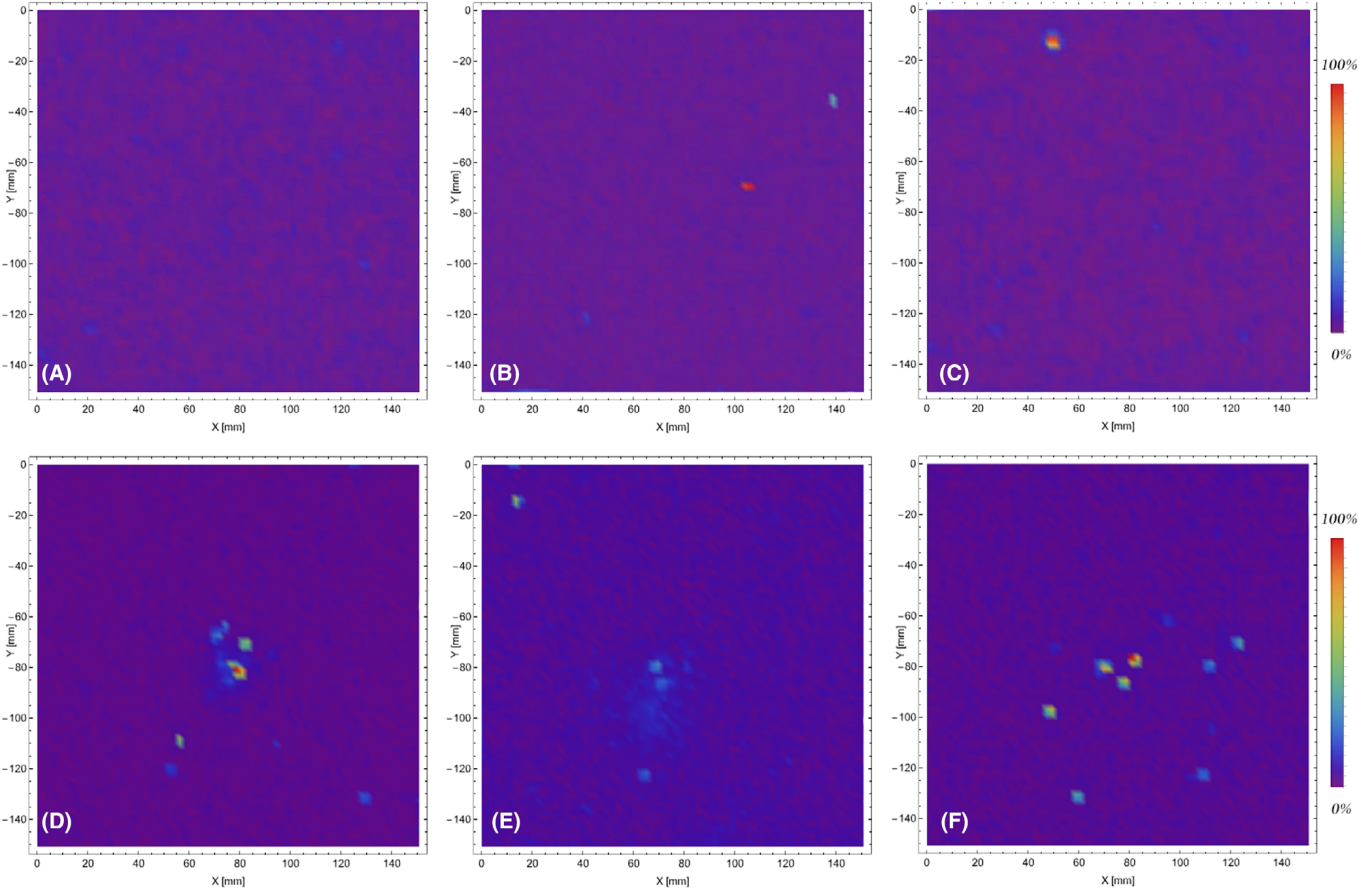
XRF maps of iron on cotton targets in rainbow false‐colors. In the upper row, specimens collected discharging the pistol without the suppressor, at the shooting distances of 5 cm (A), 20 cm (B), and 40 cm (C). In the lower row, specimens collected discharging the pistol with the suppressor mounted, at the shooting distances of 5 cm (D), 20 cm (E), and 40 cm (F). Area explored: 150 mm × 150 mm.

To verify the hypothesis reported by Romeo et al. [15] of possible production of Fe‐containing residues when using a suppressor, XRF maps of iron were then considered. While no evidence of iron was visible on targets shot with the unsilenced gun, Fe‐containing swarf, appearing as scattered bright spots in the x‐ray maps, was detected when the silenced pistol was used, up to a shooting distance of 40 cm (see Figure 5).

### 3.3 ICP‐OES

Pb, Ba, Sb, and Cu concentrations were measured on all the cotton targets and the blank specimens. For the targets hit with the suppressor assembled to the pistol, Fe and Al concentrations were also included in the ICP‐OES analyses. The amount of each element in the field blank was not significantly different from the fabric blanks (*p* < 0.05), indicating that handling contamination can be considered negligible. The fabric blank concentrations for the elements of interest are reported in Table 1.

**TABLE 1:** Concentrations of the selected elements on fabric blanks (*n* = 8; RSD < 15%).

	Pb	Ba	Sb	Cu	Fe	Al
Blank level, μg/cm^2^	0.011	0.017	<0.0035 (MQL)	0.007	0.036	<0.076 (MDL)

To evaluate the inter‐specimen variances for concentrations of the selected elements, the upper left 7 cm × 7 cm quadrant (Quadrant “1,” see Figure 3) of the three replicates of each target was analyzed. Table S3 file shows the results for the selected elements on the targets hit from a distance of 5 cm. The standard deviation for the three most abundant elements on the targets (Pb, Ba, and Cu) and for the metallic elements from the silencer (Al and Fe) is larger than the data collected by Brozek‐Mucha in a similar experiment [8]. There are two main reasons for such large variances. First, it should be noted that, of the elements selected for ICP‐OES analyses, barium comes from the primer mixture, while lead and antimony also constitute the alloy of the bullet cores. As shown for copper in a previous work [36] and confirmed here for iron (see Figure 5), elements originating from mechanical shavings of the bullets in the barrel (or in the suppressor internal cylinder) may be concentrated in a few massive metallic chips, randomly distributed on the targets. The variance in the data may have been increased by the decision to consider a single quadrant for each target. The second critical factor is the removal of the area of the target where the bullet holes are located. Bullet wipes were easily visible to the naked eye for shooting distances where soot deposits on the target were minimal—that is, ≥40 cm. At the shortest distances (5, 20 cm), the boundaries of the bullet wipes were not clearly visible among soot deposits, and it was up to the operator to decide which areas to cut: this is also supposed to increase the variance.

For a direct comparison, intra‐specimen elemental concentration variances were calculated from the analyses of three quadrants of the same replica, as reported in Table S4.

**FIGURE 6:**
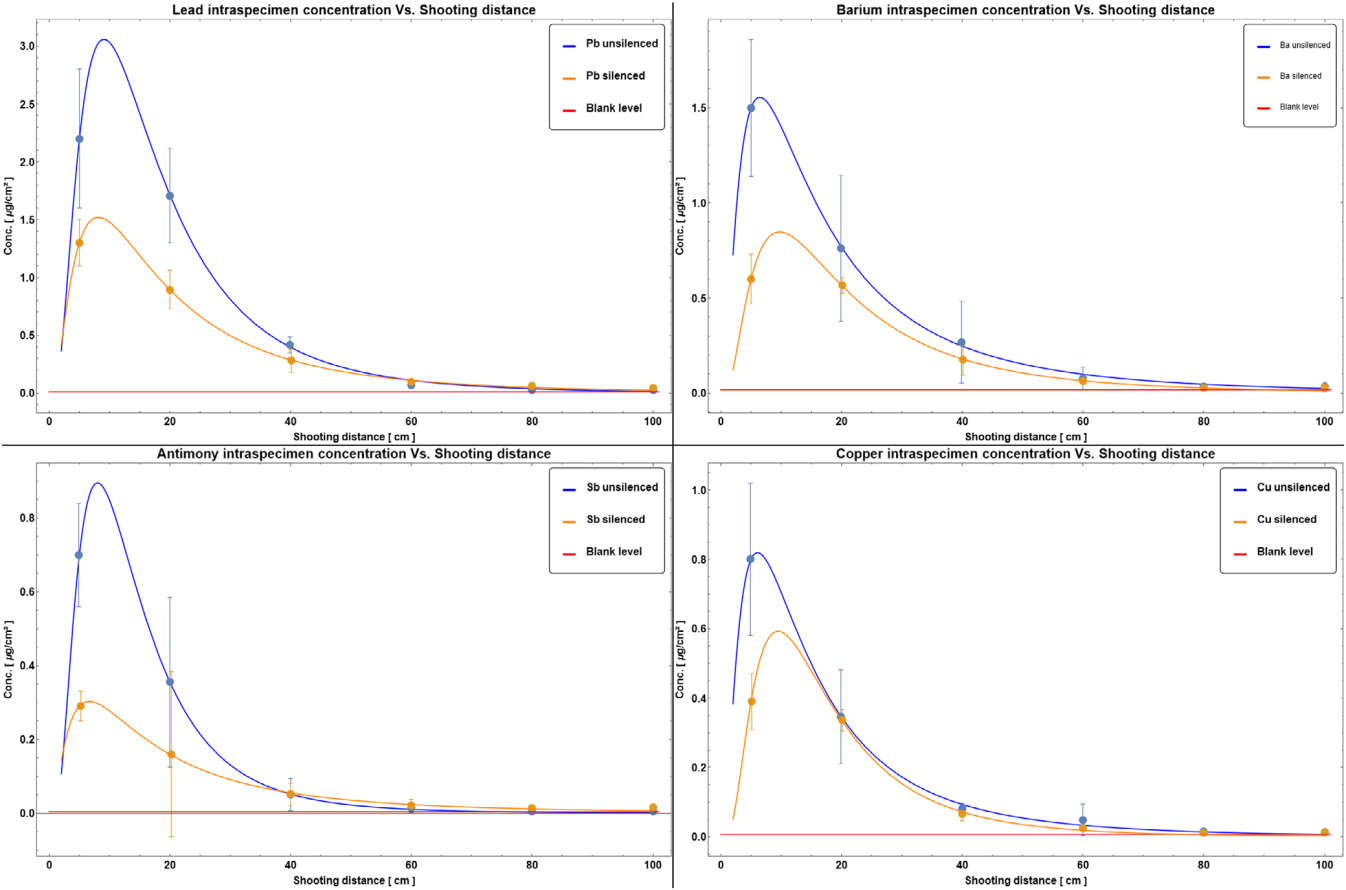
Pb, Ba, Sb, and Cu concentrations on cotton targets for all distances and both pistol configurations (unsilenced = blue marks, silenced = orange marks). Error bars correspond to 1σ. According to Brozek‐Mucha [18], maximum residue concentrations are expected to be found for shooting distances >5 cm, so all data were fit using an asymmetric log‐normal probability distribution function (solid lines in the plots).

**FIGURE 7:**
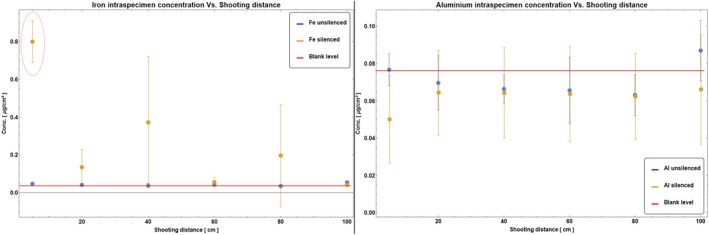
Fe and Al concentrations on cotton targets for all distances and both pistol configurations (unsilenced = blue marks, silenced = orange marks). Error bars correspond to 1σ. The red ellipse highlights the only value that differs from both the blank level and the corresponding unsilenced value with 95% confidence.

Intra‐specimen coefficients of variation were much lower than inter‐specimen ones, so standard deviations calculated for each target were used to present data in the following plots. The complete (unsilenced/silenced pistol, all shooting distances) sets of ICP‐OES results for the cotton targets are reported in Figure 6 for Pb, Ba, Sb, and Cu, and Figure 7 for Fe and Al. Data in Figure 6 show that—at a confidence level of 95%, corresponding to error bars of 2.48σ [37]—the concentrations of all elements from the discharged ammunition in the targets hit by the silenced and unsilenced gun would not be statistically different. Data in Figure 7 show that the use of a silencer, with an aluminum external cylinder, does not contribute to the Al concentration in the residue. The same can be said for the iron present in the inner cylinder of the silencer for all the shooting distances except the shortest, that is, 5 cm. In this case, a statistically significant contribution of iron to gunshot residue can be found by ICP‐OES, confirming the presence of isolated Fe‐containing particles in the fabric as previously detected by XRF analyses (see Figure 5).

## 4 CONCLUSIONS

The use of a suppressor produces different patterns of residues in the targets that are visible to the naked eye at a shooting distance of 5 cm and that are detectable by XRF maps up to 20 cm. Despite the different spatial distributions, the total amounts of the elements from the discharged ammunition did not appear to be statistically altered by the use of a suppressor. This implies that, with the chosen firearm and experimental setting, the evaluation of the determination of the shooting distance can be carried out without knowledge of the use of a silencer and without a significant error, at least by ICP‐OES analysis.

The hypothesis of a role of homemade suppressors in enriching GSR populations in iron, originating from silencer inner cylinders, was confirmed. The presence of iron deposits on the target, detected by XRF up to a shooting distance of 40 cm, is, in fact, the only indication of the actual use of a suppressor, useful for the choice of a proper method for the precise determination of the shooting distance when lead patterns on targets are considered.

## CONFLICT OF INTEREST STATEMENT

The authors declare no competing financial interests or personal relationships that could influenced the work reported in this paper.

## Supporting information


Data S1
