# Supplementary material for: Effect of mounting a sound suppressor on distribution and total amount of inorganic gunshot residue on targets
Source: J Forensic Sci. 2025 Apr 3;70(3):1165–73. doi: 10.1111/1556-4029.70025 (PMC12046103; doi:10.1111/1556-4029.70025)
Supplement: Supplementary file 1 — Data S1 [file JFO-70-1165-s001.pdf]

Supplementary Information

SEM-EDS analyses of primer gunshot residue

A scanning electron microscope model EVO-MA10 by Zeiss (Zeiss Group, Oberkochen – Germany), operating with a beam energy of 20 keV at a working distance of 8.0 mm, was used. The SEM was equipped with a LaB_6 filament and detectors for both secondary and backscattered electrons. A silicon drift detector model X-Max 50 mm^2 by Oxford (Oxford Instruments plc, Abingdon, UK) was used for X-ray collection.

Inorganic GSR particles were collected dabbing the target surface around the bullet hole using half-inch aluminium stubs having double-sided conductive tape disks on top. Collection method consisted in dabbing 30 times the area of interest.

Sound suppressor characteristics

Production of the silencer was completed the day before the shooting tests, and it was dismantled immediately afterwards. The silencer parts were smashed under a hydraulic press. The silencer used for the shooting tests was constructed according to the scheme shown in FIGURE S1.

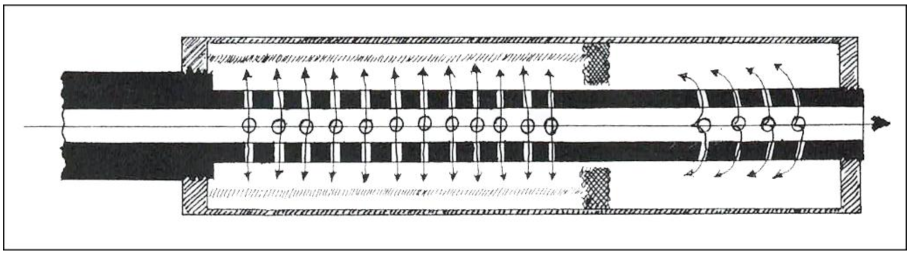

FIGURE S1   Scheme of the home-made silencer. Original image in: Ugolini A, L’Esperto Balistico Vol.1 - Le Armi, Il Munizionamento e Le Balistiche, Firenze: Editoriale Olimpia; 1978.

ICP OES analyses

Element	Calibration curve	R^2	Wavelength (nm)	Lecture mode
**Pb**	Signal (cps) = 3502,1 x Concentration (mg/L)	0,9985	220.353	Axial
**Ba**	Signal (cps) = 60902,1 x Concentration (mg/L)	0,9993	455.403	Radial
**Sb**	Signal (cps) = 1910,3 x Concentration (mg/L)	1,0000	206.833	Axial
**Cu**	Signal (cps) = 48093,6 x Concentration (mg/L)	0,9989	324.754	Axial
**Fe**	Signal (cps) = 24499,4 x Concentration (mg/L)	0,9977	259.940	Axial
**Al**	Signal (cps) = 8312,0 x Concentration (mg/L)	0,9934	308.215	Axial

TABLE S1   Calibration curves and instrumental parameters used for ICP-OES analyses.

Element	IDL (x10^{-3} mg/L)	IQL (x10^{-3} mg/L)	MDL (x10^{-3} µg/cm^2)	MQL (x10^{-3} µg/cm^2)
**Pb**	0.4	1.0	0.2	0.5
**Ba**	2.5	4.5	1.3	2.3
**Sb**	4.1	6.9	2.0	3.5
**Cu**	7.8	11.2	4.1	5.7
**Fe**	1.0	1.6	0.5	0.8
**Al**	149	182	76	93

TABLE S2 IDL-IQL, and MDL-MQL for the elements of interest.

	**Unsilenced pistol, shooting distance = 5 cm**		
	**Mean µ**	**St. deviation σ**	**CV 100 x σ/µ**
**Pb µg/cm^2**	6	2	28%
**Ba µg/cm^2**	2.1	0.7	36%
**Sb µg/cm^2**	0.69	0.08	12%
**Cu µg/cm^2**	0.9	0.3	38%
**Fe µg/cm^2**	Blank level	-	-
**Al µg/cm^2**	< MDL	-	-
	**Silenced pistol, shooting distance = 5 cm**		
	**Mean µ**	**St. deviation σ**	**CV 100 x σ/µ**
**Pb µg/cm^2**	5.6	2	33%
**Ba µg/cm^2**	1.3	0.4	34%
**Sb µg/cm^2**	0.37	0.05	12%
**Cu µg/cm^2**	0.5	0.2	35%
**Fe µg/cm^2**	0.3	0.2	66%
**Al µg/cm^2**	0.08	0.05	58%

TABLE S3 Element concentrations detected in the upper left quadrant of the three replicas of targets hit from a distance of 5 cm (inter-specimen variance).

	**Unsilenced pistol, shooting distance = 5 cm**		
	**Mean μ**	**St. deviation σ**	**CV 100 x σ/μ**
**Pb μg/cm^2**	2.2	0.6	25%
**Ba μg/cm^2**	1.5	0.3	24%
**Sb μg/cm^2**	0.7	0.1	20%
**Cu μg/cm^2**	0.8	0.2	28%
**Fe μg/cm^2**	Blank level	-	-
**Al μg/cm^2**	< MDL	-	-
	**Silenced pistol, shooting distance = 5 cm**		
	**Mean μ**	**St. deviation σ**	**CV 100 x σ/μ**
**Pb μg/cm^2**	1.3	0.2	14%
**Ba μg/cm^2**	0.6	0.1	21%
**Sb μg/cm^2**	0.29	0.04	15%
**Cu μg/cm^2**	0.39	0.08	20%
**Fe μg/cm^2**	0.08	0.01	14%
**Al μg/cm^2**	0.05	0.02	47%

TABLE S4 Element concentrations detected in quadrants 1, 2 and 3 of the targets hit from 5 cm (intra-specimen variance, referred to replica #1).
